# McMurray’s test is influenced by perimeniscal synovitis in degenerative meniscus tears

**DOI:** 10.1186/s43019-024-00242-5

**Published:** 2025-02-28

**Authors:** Yong Jun Jin, Jae-Young Park, Jun Young Chung, Sujin Noh, Hee-Woong Yun, Sumin Lim, Do Young Park

**Affiliations:** 1 Department of Orthopedic Surgery, Quanzhou First Hospital Affiliated to Fujian Medical University, Quanzhou, 362000 China; 2 Department of Orthopedic Surgery, School of Medicine, Ajou University, Suwon, Republic of Korea; 3 Cell Therapy Center, Ajou University Medical Center, Suwon, Republic of Korea; 4 Department of Biomedical Sciences, Graduate School of Ajou University, Suwon, Republic of Korea; 5 Department of Orthopaedic Surgery, CHA University, CHA Bundang Medical Center, 351 Yatap-Dong, Bundang-gu, Seongnam-si, Republic of Korea

**Keywords:** McMurray’s test, Synovitis, Degenerative meniscus tear, Mechanical symptom

## Abstract

**Background:**

McMurray’s test is a useful physical examination in determining meniscus tears, yet its sensitivity is only 38–62%. Furthermore, the relationship between degenerative meniscus tears (DMT) and mechanical symptoms during McMurray’s test is not well defined. Perimeniscal synovitis occurs in osteoarthritic (OA) knees, inducing localized symptoms such as posterior knee pain in medial meniscus posterior horn DMTs. This study aimed to determine the relationship between McMurray’s test with medial meniscus DMT and perimeniscal synovitis in patients with knee OA.

**Methods:**

We retrospectively analyzed 60 patients who underwent medial unicompartmental knee arthroplasty (UKA) with positive (*n* = 20) and negative (*n* = 40) preoperative McMurray’s tests. Preoperative magnetic resonance imaging (MRI), intraoperative gross morphology, and histological analysis of meniscus and synovium were evaluated to determine meniscal tears and perimeniscal synovitis. Univariate and multivariate regression analyses were done to determine the effects of meniscus tears and synovitis on McMurray’s test results.

**Results:**

Gross morphology of the medial meniscus (MM) showed 14 out of 20 torn menisci in the McMurray’s (+) group compared with 22 out of 40 in the (−) group, with no difference in meniscus tear severity among groups. The (+) group showed higher values of synovial thickness (*p* < 0.001) and area (*p* < 0.001) compared with the (−) group on magnetic resonance imaging (MRI). Histological analysis showed higher synovitis (*p* < 0.001) scores and expression of inflammatory markers [interleukin (IL)-1β (*p* < 0.001), IL-6 (*p* = 0.007), nerve growth factor (NGF) (*p* = 0.003), inducible nitric oxide synthase (iNOS) (*p* < 0.001)] in the perimeniscal synovium of (+) group compared with the (−) group. Multivariable logistic analysis revealed that larger synovial area [odds ratio (OR) = 1.106, *p* = 0.008] and a higher histologic synovitis score (OR = 2.595, *p* = 0.011) were independently significant predictive factors for a positive McMurray’s test.

**Conclusions:**

McMurray’s test may be influenced by perimeniscal synovitis in DMT patients. The clinical implications of our results may influence not only the interpretation of McMurray’s test but also the target tissue in treating mechanical symptoms related to meniscus tears.

**Level of evidence:**

Level II.

**Supplementary Information:**

The online version contains supplementary material available at 10.1186/s43019-024-00242-5.

## Introduction

McMurray’s test is one of the most commonly performed physical examinations for diagnosing meniscal tears, being especially useful in detecting posterior meniscus tears [1, 2]. While simple to perform, the reliability of this test has often been in question, with reported sensitivities of 38–62%, specificities of 57–90%, and positive predictive values of 61–84% for medial meniscus (MM) tears [3]. The relatively low sensitivity of McMurray’s test may lead to missed diagnosis for posterior meniscus tears.

The relationship between meniscus tears and mechanical symptoms during McMurray’s test is questionable. Tears situated in the inner meniscus may be asymptomatic during mechanical insults, owing to nerve supply of the meniscus, which is limited to the outer 10–25% [4–6]. Furthermore, degenerative meniscus tears (DMT) with mechanical symptoms often respond to nonsurgical treatments such as physical therapy, oral antiinflammatory analgesics, and intraarticular injection [7–9]. Arthroscopic partial meniscectomy (APM), on the other hand, does not always result in relief of mechanical joint symptoms and functional improvement [10–12]. Therefore, it is possible that DMTs are not the primary cause of positive results during McMurray’s test.

The meniscus is surrounded by perimeniscal synovial tissue at the outer edge. Synovitis is a well-known factor in osteoarthritis (OA) disease progression and pain [13, 14]. The perimeniscal synovium adjacent to DMTs expressed higher levels of inflammatory markers such as tumor necrosis factor alpha (TNF-α) and IL-6 in a previous study performed in APM patients [15]. We hypothesized that the mechanical symptoms during McMurray’s test is associated with perimeniscal synovitis. We aimed to evaluate the relationship of McMurray’s test results with medial DMTs and perimeniscal synovitis by gross morphology, histology, and MRI data in knee OA patients.

## Patients and methods

### Study design

**Fig. 1:**
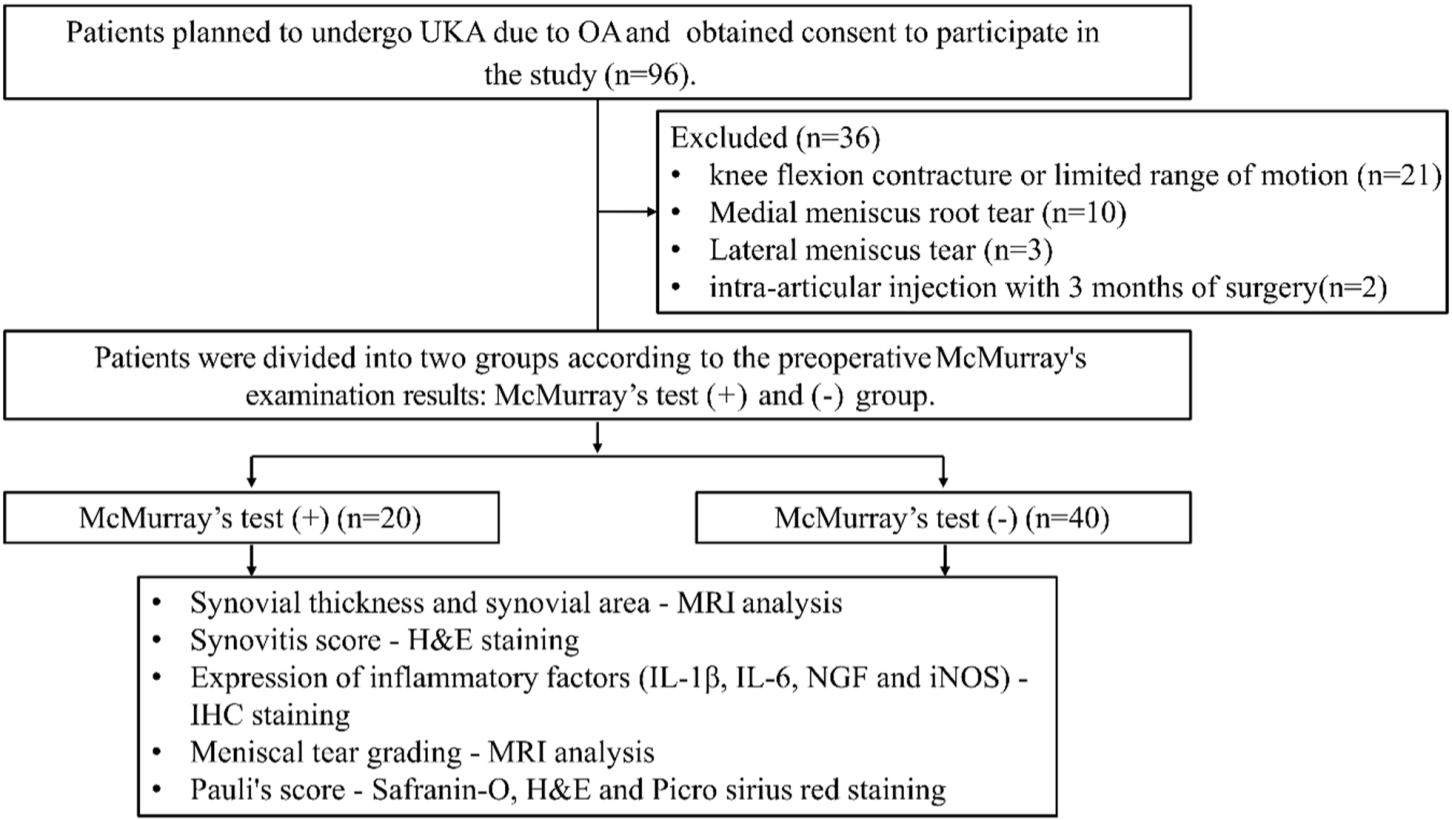
Study flowchart

This study was designed as a case–control study. All data collection and analysis were completed with the approval of our Institutional Review Board (AJOUIRB-SM-2023-155). A total of 60 consecutive patients with primary knee Kellgren–Lawrence (K–L) grade 2, 3 OA undergoing medial unicompartmental knee arthroplasty (UKA) at our institution from January 2020 to December 2022 were included. Patients with subchondral insufficiency fracture of the knee (SIFK)/spontaneous osteonecrosis of the knee (SONK) were also included. Patients with inflammatory joint disease, posttraumatic OA, history of previous knee surgery, lateral meniscus tear, MM root tear, and ligament deficiencies were excluded. Patients with knee flexion contracture (> 10°) or limited range of motion (knee flexion < 135°) were also excluded. Patients were divided into those with positive (+) and negative (−) McMurray’s test results. The key outcome indicators included gross morphology, magnetic resonance imaging (MRI), and histological data regarding meniscus and perimeniscal synovium (Fig. 1).

### McMurray’s test protocol

McMurray’s test was performed the day before surgery for every patient by the senior author (Do Young Park). Briefly, the patient lay in a supine position with the knee fully bent. The examiner’s proximal hand held the knee while palpating the joint line, thumb on one side and fingers on the other, and the distal hand held the sole of the foot. From a position of maximal flexion, the knee is extended under tibial internal/external rotation and varus/valgus stress. A test was ruled positive if the test resulted in pain along the joint line, snapping, an audible locking of the knee [16].

### Meniscus tear gross morphology

Meniscus tears found during surgery were recorded after gross examination, and tear patterns were classified according to the International Society for Arthroscopy, Knee Surgery, and Orthopedic Sports Medicine (ISAKOS) classification by two separate orthopedic surgeons blinded to preoperative patient data [17].

### MRI protocol

All MRI studies were carried out on a 1.5-T MR unit (Discovery MR750W; GE Healthcare, Waukesha, WI) using a transmit–receive quadrature knee coil (GE Healthcare) within 6 months of surgery. MRI scans consisted of sagittal T1 FS (TR/TE = 710/9, slice = 3 mm, field of view = 160 mm), sagittal T2 FS (TR/TE = 3824/75, slice = 3 mm, field of view = 160 mm), sagittal PD FS (TR/TE = 1800/25, Slice = 3 mm, field of view = 160 mm), coronal T2 FS (TR/TE = 2331/70, slice = 3 mm, field of view = 160 mm), and axial PD FS (TR/TE = 1800/25, slice = 3 mm, field of view = 160 mm).

### MRI grading

**Fig. 2:**
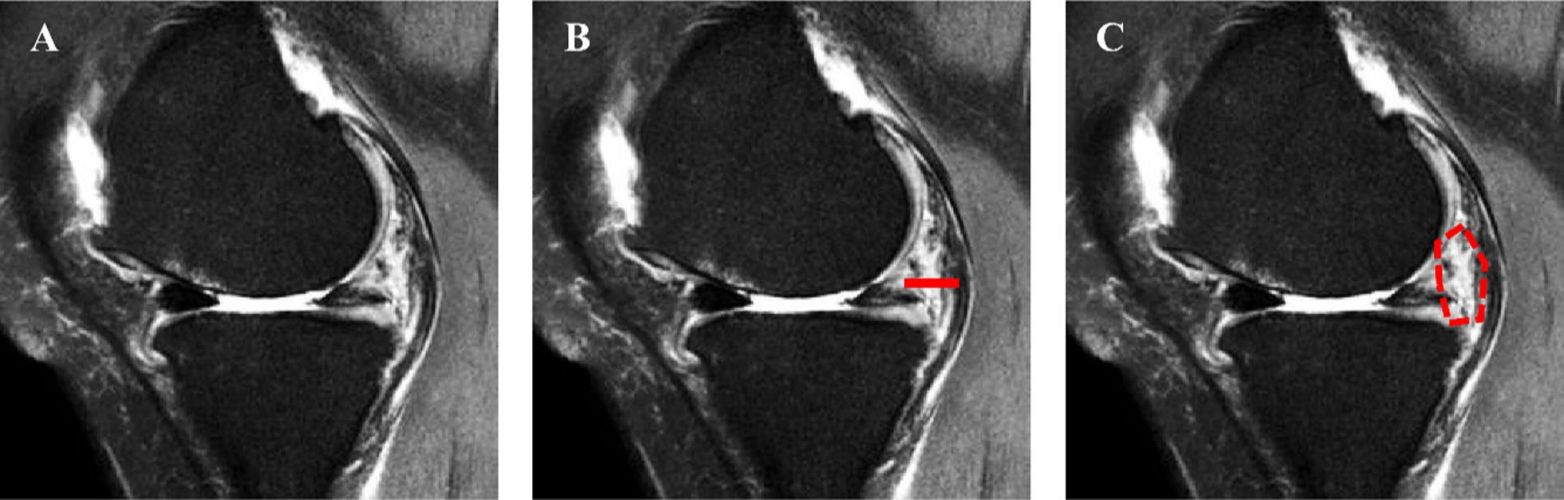
Schematic of measuring MRI parameters. **A** Medial meniscal tear grade was measured on sagittal MRI T2 images. **B** Synovial thickness measured in the same sagittal MRI T2 image. **C** Synovial hyperintense areas measured in the same sagittal MRI T2 image

The grading of DMTs was performed according to the previously published meniscus grading system (Fig. 2A, Supplementary Table 1) [18], by two independent orthopedic surgeons.

### Radiological analysis

Standing anterior–posterior (AP) of the knee and MRI of the knee were routinely performed prior to surgery. The K–L grading system was used to assess preoperative knee OA from an AP perspective. Lower limb alignment was measured as the angle formed between the mechanical axes of the femur and tibia on whole-leg standing AP radiographs. Perimeniscal synovial tissue was assessed in sagittal T2 image at the level where the medial meniscus appears longest in an axial image. For synovial thickness, we measured the distance from the posterior border of the MM to the semimembranosus tendon, parallel to the tibial slope (Fig. 2B). For the synovium area, the area of high signal intensity between the posterior border of the MM to the semimembranosus tendon was measured (Fig. 2C) [19–21]. All radiographic measurements were performed using the Picture Archiving and Communication Systems (PACS: Carestream Health, Rochester, New York, USA). The final data were determined by calculating the average of the results obtained by two experienced radiologists who performed the MRI image analysis in a blinded manner.

### Histology and Immunohistochemistry analysis

During UKA, the MM was carefully dissected off the knee joint to include 2–4 mm of perimeniscal synovium in the posterior horn without the use of electrocautery. The MM and perimeniscal synovium were evaluated by gross examination and histology. Histologic specimens were fixed in 10% neutral buffered formalin (NBF) for 3 days at room temperature. Specimens were subsequently embedded in paraffin wax. Sections with a thickness of 4 μm were made and stained with Safranin O/Fast green, hematoxylin and eosin (H&E), and Picrosirius Red (PR). Tissue sections were observed under a light microscope (E600; Nikon, Tokyo, Japan) and evaluated by two investigators who were not involved in this study. Meniscal tissue degeneration was assessed by meniscus degenerative scoring system, which has a total of grades 1–4, with lower grades indicating less degeneration (Supplementary Table 2) [22]. The synovial tissue was evaluated by a previously published synovitis scoring system on a scale of 0–9, with lower scores indicating less degeneration (Supplementary Table 3) [23]. For immunohistochemical staining of synovial tissue, anti-IL-1β (1:200, Abcam, ab283818, Cambridge, UK), anti-IL-6 (1:200, Abcam, ab214419, Cambridge, UK), anti-NGF (1:200, Abcam, ab51928, Cambridge, UK), and anti-iNOS (1:200, Abcam, ab3523, Cambridge, UK) antibodies and an immunohistochemical detection kit (GBI labs, Bothell, WA, USA) were used. The experimental procedure was as follows; slides were treated with 3% H_2_O_2_ for 10 min to eliminate endogenous peroxidase from red blood cells. After 10 min of incubation with pepsin solution, slides were changed to blocking buffer for 60 min. The primary antibody was then treated overnight, followed by incubation with biotinylated secondary antibody for 2 h. Streptavidin-conjugated peroxidase reagent was treated for 30 min and then reacted with diaminobenzidine (DAB) chromogen to generate the signal. Finally, slides were counterstained using Harris’ hematoxylin (Sigma-Aldrich, MO, USA). Subsequently, stained samples were quantified using ImageJ software. The final score was determined by calculating the average of the results obtained by two investigators who conducted the histological scoring in a blinded manner.

### Statistical analysis

In this study, all quantitative datasets are expressed as mean ± standard deviation (SD). The data were analyzed using GraphPad Prism 8 (GraphPad Software, La Jolla, CA, USA) and R 3.6.3 (R Foundation for Statistical Computing, Vienna, Austria). The radiological and histologic outcomes were analyzed through the *t*-test after confirming normality with the Shapiro–Wilk test. For noncontinuous variables, either the chi-squared test or Fisher’s exact test was performed. Univariable and multivariable regression analyses were performed to determine the predictive factors for the positive McMurray’s test. The level of significance was set at *p* < 0.05 (*p < 0.05, **p < 0.01, ***p < 0.001).

### Intraobserver and interobserver reliability

Intraobserver and interobserver reliability were assessed using intraclass correlation coefficients (ICC) for continuous variables, while categorical features were evaluated using linear weighted kappa statistics. Two observers conducted their assessments in a blinded fashion, with the first observer performing a second measurement after 4 weeks to assess intraobserver reliability. For categorical variables, the first measurement from the first observer was used for analysis, while for continuous variables, the average of the two observers’ measurements was utilized. For ICC and kappa, values below 0.2 were considered to indicate poor agreement, values between 0.21 and 0.40 as fair, 0.41 to 0.60 as moderate, 0.61 to 0.80 as good, and values above 0.80 as excellent [24].

For MRI measurements, intraobserver ICC and kappa values ranged from 0.886 to 0.970, and interobserver values ranged from 0.786 to 0.932. For histological and immunohistochemical assessments, intraobserver ICC values ranged from 0.908 to 0.967, and interobserver values ranged from 0.798 to 0.956, showing good to excellent consistency.

## Results

### Demographic data

All demographic data were comparable, and there were no significant differences between the two groups (Table 1). Six and 11 knees were diagnosed with SIFK in the medial femoral condyle in the McMurray’s (+) and (−) group, respectively, with no statistical difference (*p* > 0.99). The lower limb alignment was varus 5.4 ± 2.3 versus varus 4.6 ± 1.9 in the (+) and (−) group respectively, showing no statistical difference (*p* = 0.912). Overall, the McMurray’s test sensitivity was 47.4%, the specificity was 74.1%, and the positive predictive value was 65%.

**Table 1:** Demographic data

Variable	McMurray’s (+)	McMurray’s (−)	*p*-Value
Number	20	40	
Age (years)	64.45 ± 8.0	65.1 ± 8.1	0.572
Sex, M/F	2/18	3/37	> 0.99
BMI (kg/m^2^)	25.3 ± 2.0	25.2 ± 2.8	0.984

BMI, body mass index

### Medial meniscus tear analysis

**Fig. 3:**
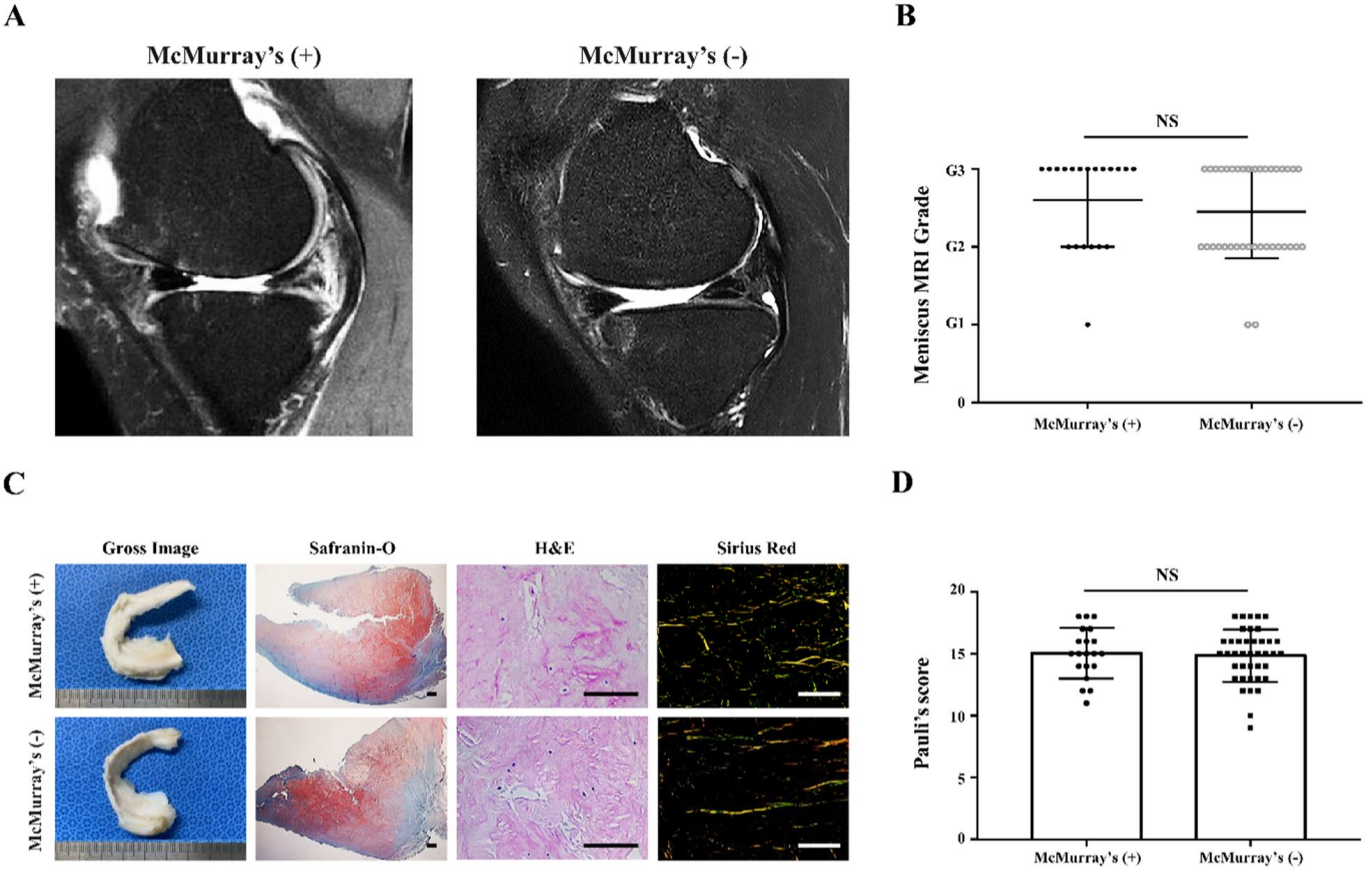
MRI and histological evaluation of the MM. **A** Sagittal MRI T2 images of MM in (+) and (−) group. **B** Grading of MM tear in MRI T2 images of (+) and (−) group. **C** Morphological and histological analysis (Safranin-O, H&E, and PR staining) of meniscal tissue in (+) and (−) group. **D** Pauli’s score of meniscal tissues in (+) and (−) group. Scale bar = 100 μm. Data are presented as mean and standard deviation. ns = not significant

Gross morphology of the MM showed 14 out of 20 torn menisci in the McMurray’s (+) group compared with 22 out of 40 in the (−) group. All tear locations found in this study were in the MM posterior horn (MMPH). Tear morphology consisted of 8 and 14 horizontal tears, together with 6 and 8 complex tears for the (+) and (−) group, respectively (*p* = 0.969). As for MRI, sagittal MRI images showed tears in the MMPH in all knees with MM tears (Fig. 3A). Thirteen and 20 knees showed a hyperintense liner signal extending to the articular surface of the tibia in the (+) and (−) group, respectively, in the sagittal T2 images. MRI grading results showed that there was no statistical difference between the two experimental groups (Fig. 3B, *p* = 0.601). Histological degeneration of the menisci showed no statistical difference between the two groups (Fig. 3C, D, *p* = 0.726).

### Perimeniscal synovium analysis

**Fig. 4:**
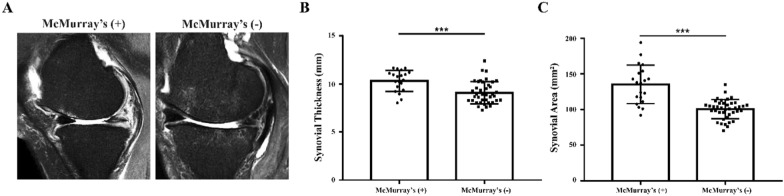
MRI assessment of synovial thickness and area. **A** Sagittal MRI T2 images of synovium tissue in (+) and (−) group. **B**, **C** Quantitative analysis of synovial thickness and synovial hyperintensity area in sagittal MRI T2 images of (+) and (−) group. Data are presented as mean and standard deviation. ***p < 0.001

In the sagittal MRI T2 images, we found that the area of perimeniscal synovium was wider in the (+) group than in the (−) group (Fig. 4A). The results of synovial thickness measurement showed that the synovial thickness of the (+) group (10.3 ± 1.1 mm) was significantly higher than that of the (−) group (9.1 ± 1.2 mm), with a statistical difference (Fig. 4B, *p* < 0.001). In the same MRI T2 image, the synovium area measurement results showed that the synovial area of the (+) group (135.1 ± 27.1 mm^2^) was significantly higher than that of the (−) group (100.4 ± 13.5 mm^2^) (Fig. 4C, *p* < 0.001) (Table 2).

**Table 2:** Radiologic and histology outcomes in two groups

Measurement	McMurray’s (+)	McMurray’s (−)	*p*-Value
Meniscus grade 1/2/3	1/6/13	2/18/20	0.601
Meniscus tear morphology	14	22	0.409
Meniscus histology	15.1 ± 2.0	14.9 ± 2.4	0.726
Synovial thickness (mm)	10.3 ± 1.1	9.1 ± 1.2	< 0.001
Synovial area (mm^2^)	135.1 ± 27.1	100.4 ± 13.5	< 0.001
Synovitis score	6.0 ± 1.2	4.5 ± 1.2	< 0.001

**Fig. 5:**
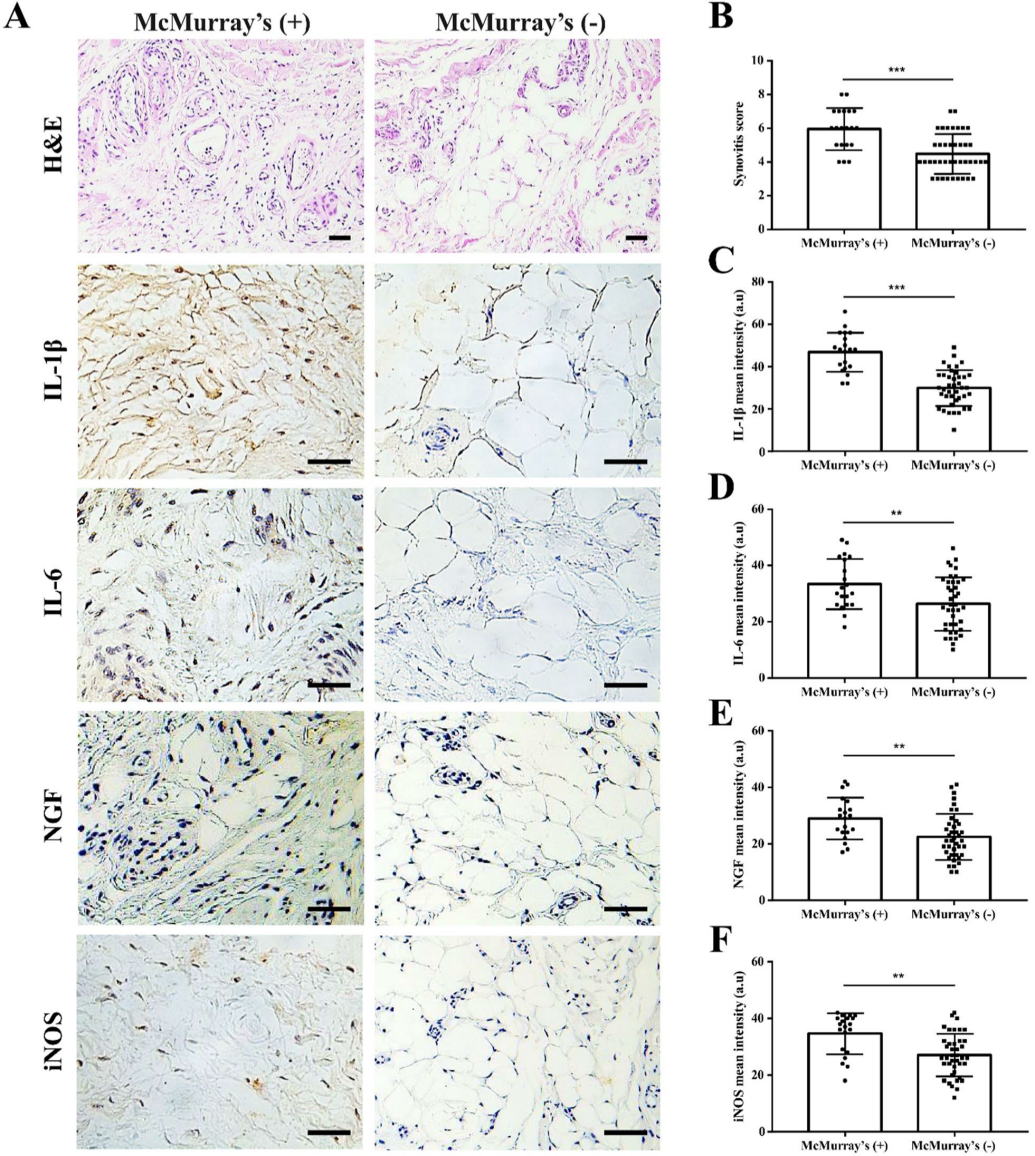
Histological and immunohistochemical assessment of synovial inflammation. **A** H&E and immunohistochemical staining images of synovium tissue in (+) and (−) group. **B** Synovitis score of synovial tissue in (+) and (−) group. **C**–**F** Quantitative analysis of the staining intensity of inflammatory factors in synovial tissue of (+) and (−) group. Scale bar = 100 μm. Data are presented as mean and standard deviation. **p < 0.01, ***p < 0.001

Histology of the perimeniscal synovium showed synovial layer hyperplasia and stromal cell density increase, with more inflammatory cells in the McMurray’s test (+) group compared with the (−) group. Immunohistochemical staining images showed increased synovial IL-1β, IL-6, NGF, and iNOS expression in the (+) group compared with the (−) group (Fig. 5A). Overall, the histological synovitis score was significantly higher in the (+) group compared with the (−) group (Fig. 5B, *p* < 0.001). Quantitative analysis of the staining intensity of IL-1β, IL-6, NGF, and iNOS in the synovium showed that the staining intensity of the (+) group was higher than (−) group (Fig. 5C–F, *p* < 0.001, *p* = 0.007, *p* = 0.003, *p* < 0.001).

### Univariable and multivariable logistic regression analysis

The univariable logistic regression analysis demonstrated that increased synovial thickness (OR = 2.430, *p* = 0.001), larger synovial area (OR = 1.105, *p* < 0.001), and a higher histologic synovitis score (OR = 2.611, *p* < 0.001) were significantly associated with positive McMurray’s test. The multivariable logistic analysis revealed that larger synovial area (OR = 1.106, *p* = 0.008) and a higher histologic synovitis score (OR = 2.595, *p* = 0.011) were independently significant predictive factors for positive McMurray’s test (Table 3).

**Table 3:** Univariable and multivariable logistic regression analysis of predictive factors for positive McMurray’s test

Variable	Univariable			Multivariable		
	Odds ratio	95% CI	*p*-Value	Odds ratio	95% CI	*p*-Value
Age	0.990	0.925–1.059	0.765			
K–L grade (ref: 2)	1.000	0.310–3.226	> 0.99			
BMI (kg/m^2^)	1.009	0.815–1.250	0.933			
Sex (ref: male)	0.730	0.112–4.762	0.742			
Meniscus tear-M (ref: no tear)	1. 909	0.610–5.977	0.267			
Meniscus tear-MRI (ref: no tear)	1.857	0.613–5.626	0.274			
Meniscus histology	1.048	0.806–1.363	0.724			
Synovial thickness (mm)	2.430	1.424–4.150	0.001	0.970	0.374–2.516	0.950
Synovial area (mm^2^)	1.105	1.047–1.167	< 0.001	1.106	1.027–1.191	0.008
Synovitis score	2.611	1.521–4.480	< 0.001	2.595	1.242–5.421	0.011

CL, confidence interval; K–L, Kellgren–Lawrence; BMI, body mass index; ref, reference; M, morphology

## Discussion

The aim of this study was to find the relationship between McMurray’s test and perimeniscal synovitis via gross morphology, MRI, and histology in knees with DMTs. The most important finding of this study is that McMurray’s test results correlated with perimeniscal synovitis. McMurray’s test (+) patients showed increased synovitis features of inflammation and pain such as IL-, IL-6, NGF, and iNOS compared with McMurray’s test (−) patients.

McMurray’s test was first described by Thomas Porter McMurray in 1942 as a physical examination to detect meniscal tears [25]. McMurray’s test has gone through many modifications since the original description, with the inclusion of pain in a specific area or apprehension [16, 26]. It is postulated that the torn meniscus fragment can be manipulated between the femur and tibia, possibly trapping a meniscus fragment to lock the knee in a certain position [27]. While McMurray’s test is one of the most commonly performed physical examinations to detect meniscus tears, the overall reported sensitivity is poor, ranging from 38% to 62% [3]. The sensitivity was higher in patients with a typical meniscal injury history and lower in patients with chronic knee pain [28]. False positive results may arise from cartilage damage, synovitis, and ligamentous lesions, commonly found in knees with DMTs. The lack of knowledge regarding the test’s exact mechanism and affected anatomical structures lead to the overall poor sensitivity of the McMurray’s test.

Perimeniscal synovitis may be the key factor associated with pain and discomfort experienced during McMurray’s test with DMTs, as shown in our data. As we have hypothesized, synovitis severity and perimeniscal synovial area were significant factors for a positive test. Synovial thickness was significantly associated with (+) results on univariate analysis but not on multivariate analysis (Table 3). The mechanical symptoms during McMurray’s test may be a result of synovial impingement, which is more likely to occur if the synovial area is large. Synovial thickness, which was the distance between the posterior border of MM to the semimembranous tendon, may be influenced by confounding factors such as subtle differences in knee flexion angles, position of semimembranosus, and MM. From an anatomical aspect, it is highly unlikely that the meniscus tear site, especially when located in the inner two-thirds of the meniscus, is the culprit of symptoms during McMurray’s test. Myelinated and free nerve endings are limited to the outer one-third of the meniscus, while nerve fibers are widely distributed in the adjacent synovium [29, 30]. This innervation pattern also remains similar in DMTs [31, 32]. Furthermore, mechanical symptoms and McMurray’s test results of the knee do not always correlate with presence of DMTs. Sihvonen et al. reported that, of the 146 patients who had received arthroscopic surgery for DMTs, only 21% reported positive preoperative McMurray’s test results [10, 33]. The postoperative severity and frequency of mechanical symptoms were similar between patients who had received APM, where the torn meniscus is removed, and sham surgery [10, 33]. In contrast, previous studies report alleviation of mechanical symptoms in patient with DMTs through antiinflammatory medication and physical therapy, where the tears are left in place [34–36]. Considering the above evidence, we believe that perimeniscal synovitis may affect McMurray’s test results. An enlarged and inflamed perimeniscal tissue may be more likely to be under biomechanical stress during McMurray’s test.

The strength of our study lies in the study design and samples analyzed. We utilized meniscus and synovium samples from patients undergoing UKA, where tissues can be safely harvested during the surgical procedure. We did not include knees undergoing total knee arthroplasty (TKA), since such knees often have knee contracture, limited range of motion, and macerated MM, which make McMurray’s test impossible to perform in many cases. Knees undergoing UKA, in contrast, have several advantages for this study. First of all, such knees have a relatively homogeneous degree of radiologic OA limited to the medial compartment. Secondly, such knees are more likely to present with a varied degree of DMTs compared with knees undergoing TKA. Thirdly, knees undergoing UKA are more likely to have full range of knee motion compared with knees undergoing TKA, suitable for McMurray’s test. Lastly, factors affecting synovitis are relatively controlled in knees undergoing UKA, since the surgical indication is limited to medial compartment OA. Factors known to aggravate synovial inflammation, such as joint instability, mechanical stress, metabolic changes in the joint, and cartilage fragments deposition, were similar among knees analyzed, given the relatively narrow indication of UKA [37–39].

The clinical significance of this study is as follows: Our data may explain the varied sensitivity of the McMurray’s test. Clinicians should take into account that the perimeniscal synovitis status may affect the test’s outcome. Furthermore, our study may affect the decision-making process of APM for DMTs. Current practice guidelines, despite increased evidence against APM in treating mechanical knee symptoms for DMTs, continue to state that patients with mechanical symptoms may benefit from APM [40]. Ji et al. reported the utility of McMurray’s test to determine “symptomatic” DMTs and favorable postoperative outcomes after APM [41]. In light of our findings, APM likely decreases the overall area of tissue impingement during McMurray’s test, thereby offering more favorable short-term outcomes. If, however, perimeniscal synovitis is the key determinant for a positive test, as our data suggest, McMurray’s test results may change with antiinflammatory drugs, physical therapy, or even arthroscopic lavage [10, 33]. Further clinical trials are required to evaluate the course of McMurray’s test results and relationship with subjective mechanical symptoms in patients with DMTs.

There are some limitations to this study. First of all, there may be some confounding factors within the knee that affect the preoperative McMurray’s test results other than DMTs and perimeniscal synovitis. While the narrow indication of UKA allow for the analysis of a relatively homogeneous group of DMT patients, numerous other factors such as cartilage status, OA phenotype, and anatomic factors may influence McMurray’s test results and synovitis. Secondly, the obtained results may not be applicable in meniscus tears other than MM posterior horn DMTs. Younger patients with isolated meniscus tears in otherwise normal knees may have more sensitive McMurray’s test results and greater improvements of mechanical symptoms after APM [42–44]. Further clinical studies are required to analyze the role of perimeniscal synovitis in different subset of meniscal tear patients.

## Conclusions

McMurray’s test may be influenced by perimeniscal synovitis in DMT patients. The clinical implications of our results may influence not only the interpretation of McMurray’s test but also the target tissue in treating mechanical symptoms.

## Supplementary Information

Supplementary Material 1.

## Data Availability

The datasets used and/or analyzed during the current study are available from the corresponding author upon reasonable request.
