# Supplementary material for: McMurray’s test is influenced by perimeniscal synovitis in degenerative meniscus tears
Source: Knee Surg Relat Res. 2025 Feb 28;37:9. doi: 10.1186/s43019-024-00242-5 (PMC11871708; doi:10.1186/s43019-024-00242-5)
Supplement: Supplementary file 1 — Supplementary Material 1. [file 43019_2024_242_MOESM1_ESM.docx]

**Supplemental Table 1 MRI meniscus tear grading system**

| Grade | MRI finding | Interpretation |
| --- | --- | --- |
| Grade 1 | Rounded or amorphous signal that does not disrupt an articular surface | Intrasubstance degeneration |
| Grade 2 | Linear signal that does not disrupt an articular surface | Intrasubstance degeneration |
| Grade 3 | Rounded or linear signal that disrupts an articular surface | Meniscal tear |

**Supplemental Table 2 Meniscus degenerative scoring system**

| I | Surface including lamellar layer | Score |
| --- | --- | --- |
| I-1 | Femoral side: |  |
|  | A Smooth | 0 |
|  | B Slight fibrillation or slightly undulating | 1 |
|  | C Moderate fibrillation or markedly undulating | 2 |
|  | D Severe fibrillation or disruption | 3 |
| I-2 | Tibial side: |  |
|  | A Smooth | 0 |
|  | B Slight fibrillation or slightly undulating | 1 |
|  | C Moderate fibrillation or markedly undulating | 2 |
|  | D Severe fibrillation or disruption | 3 |
| I-3 | Inner border: |  |
|  | A Smooth | 0 |
|  | B Slight fibrillation or slightly undulating | 1 |
|  | C Moderate fibrillation or markedly undulating | 2 |
|  | D Severe fibrillation or disruption | 3 |
| II | Cellularity |  |
|  | A Normal | 0 |
|  | B Diffuse hypercellularity | 1 |
|  | C Diffuse hypo/acellular regions | 2 |
|  | D Hypocellularity (empty lacuna, pycnotic cells) | 3 |
| III | Collagen organization/alignment and fiber organization |  |
|  | A Collagen fiber organized, homogenous eosinophilic staining of extracellular matrix | 0 |
|  | B Collagen fibers organized, diffuse foci of hyaline or mucinous degeneration | 1 |
|  | C Collagen fibers unorganized, confluent foci or bands of hyaline or mucinous degeneration, fraying | 2 |
|  | D Collagen fibers unorganized, fibrocartilaginous separation (edema, cyst formation), severe fraying and tears | 3 |
| IV | Matrix staining (Safranin-O-Fast Green) |  |
|  | A None | 0 |
|  | B Slight | 1 |
|  | C Moderate | 2 |
|  | D Strong | 3 |
| Score 0–4 = Grade 1; Score 5–9 = Grade 2; Score 10–14 = Grade 3; Score 15–18 = Grade 4 | | |

**Supplemental Table 3 Synovitis scoring system**

| I | The synovial stroma show normal cellularity |  |
| --- | --- | --- |
|  | A The lining cells form one layer | 0 |
|  | B The lining cells form 2-3 layers | 1 |
|  | C The lining cells form 4-5 layers, few multinucleated cells might occur | 2 |
|  | D The lining cells form more than 5 layers, the lining might be ulcerated and multinucleated cells might occur | 3 |
| II | Density of the resident cells |  |
|  | A The synovial stroma shows normal cellularity | 0 |
|  | B The cellularity is slightly increased | 1 |
|  | C The cellularity is moderately increased, multinucleated cells might occur | 2 |
|  | D The cellularity is greatly increased, multinucleated giant cells, pannus formation and rheumatoid granulomas might occur | 3 |
| III | Inflammatory infiltrate |  |
|  | A No inflammatory infiltrate | 0 |
|  | B Few mostly perivascular situated lymphocytes or plasma cells | 1 |
|  | C Numerous lymphocytes or plasma cells, sometimes forming follicle-like aggregates | 2 |
|  | D Dense band-like inflammatory infiltrate or numerous large follicle-like aggregates | 3 |
| Score 0-1 = No synovitis; Score 2-4 = Low-grade synovitis; Score 5-9 = High-grade synovitis | | |
